# Rapamycin prevents retinal neovascularization by downregulation of cyclin D1 in a mouse model of oxygen-induced retinopathy

**DOI:** 10.1186/s12886-020-1325-5

**Published:** 2020-02-03

**Authors:** Feng Jiang, Ying Wang, Shufang Du, Heng Jin, Jindong Han

**Affiliations:** 1 Department of Ophthalmology, Tianjin Medical University General Hospital, Tianjin, China; 2 Department of Ophthalmology, Shenyang Aier Eye Hospital, Shenyang City, China; 3 Department of Ophthalmology, Shanxi Eye Hospital, Taiyuan City, China; 4 Department of Emergency Medicine, Tianjin Medical University General Hospital, Tianjin, China; 5 Department of Vitreous and Retina, Tianjin Medical University Eye Hospital, Tianjin, China

**Keywords:** Retinal neovascularization, Prevention, Rapamycin, Cell cycle, Animal experiment

## Abstract

**Background:**

Rapamycin (RAPA) is a potent angiogenic inhibitor and the aim of this study is to identify the inhibitory effect of RAPA on retinal neovascularization (RNV) in experimental oxygen-induced retinopathy (OIR).

**Methods:**

Forty-two 7-day-old C57BL/6 J mice were randomly divided into normoxia control group (14 mice), OIR group (14 mice), and rapamycin (RAPA) group. OIR model was induced in OIR and RAPA group. Vehicle and RAPA (2 mg/kg/d) was injected intraperitoneally daily from postnatal day 12 (P12) in OIR and RAPA groups, respectively. RNV was evaluated using fluorescence angiography and histopathology on P17. Non-perfused areas of retina were analyzed by Image-Pro plus 6.0 software. Retinal expression of cyclin D1 was detected both at mRNA and protein levels.

**Results:**

RAPA treatment significantly decreased RNV, non-perfused areas and number of endothelial cell nuclei breaking through the internal limiting membrane (ILM) in OIR mice. Moreover, RAPA decreased activation of cyclin D1 in retina caused by OIR.

**Conclusion:**

RAPA can inhibit RNV by downregulating the expression of cyclin D1, which indicates its therapeutic potential in treating RNV-related diseases.

## Background

Retinal neovascularization (RNV) is one of the leading causes of blindness in a wide range of ocular diseases, such as diabetic retinopathy (DR), age-related macular degeneration (AMD), central and branch retinal vein occlusion (CRVO and BRVO), retinopathy of prematurity (ROP) and so on [1]. Angiogenesis, the process responsible for RNV, brings about cellular and morphological changes, including endothelial cells (ECs) activation [2].

Mammalian target of rapamycin (mTOR) protein plays key roles in the activation of quiescent ECs [3], and mTOR inhibitors induce G1 cell cycle arrest [4], resulting in inhibition of ECs proliferation, migration and tube formation [5, 6]. Our previous study showed that mTOR inhibitor, rapamycin (RAPA), could inhibit the proliferation of Rhesus retinal vascular endothelial cells by downregulating cyclin D1 in vitro [7].

In the current study, our intent was to demonstrate that RAPA prevents RNV in an oxygen-induced retinopathy (OIR) model.

## Methods

### Animals

The experimental procedures performed on mice were approved by Tianjin Medical University, Laboratory Animal Care and Use Committee; and the study protocol followed the Association for Research in Vision and Ophthalmology (ARVO) for the Use of Ophthalmic Animals. Forty-two 7-day-old C57BL/6 J mice (Academy of Military Medical Science, Beijing, China) were randomly divided into normoxia control group (CON) (14 mice), OIR group (14 mice), and RAPA group (14 mice). OIR model was induced in OIR and RAPA groups according to method described by Smith et al. [8]. Briefly, 7-day-old C57BL/6 mice were exposed to 75% oxygen for 5 days, then abruptly returned to room air. Mice in RAPA group were treated with RAPA (dissolved in 2% carboxymethylcellulose, 2 mg/kg/d) by intraperitoneal injection every day from postnatal day 12(P12) to P17. And mice in OIR group were treated with the same volume of the vehicle (carboxymethylcellulose). Mice were fed commercial mouse food and were allowed access to water freely in a room with a 12 h light/12 h dark cycle. The experiments were performed on P17.

### Retinal flat mounts

Retinal flat mounts were used to show the non-perfused areas and neovascularization in retina. Four animals from each of the three groups were anesthetized with pentobarbital sodium (50 mg/Kg) by intraperitoneal injection. Mice were perfused with fluorescein isothiocyanate (FITC)-dextran (Sigma, St. Louis, MO, USA) through left ventricle. Then eyes were enucleated after euthanasia (intraperitoneal injection with pentobarbital sodium, 800 mg/Kg) and fixed in 4% paraformaldehyde at 4 °C for 12 h. Retinas were isolated, flat-mounted on glycerol/gelatin-coated glass slides, and viewed by fluorescent microscope (Zeiss, Oberkochen, Germany), and photographed. Areas of retinal nonperfusion were quantified by Image-Pro plus 6.0 analysis software for statistical analysis.

### Histopathology

The severity of RNV was quantified by counting the number of vascular cell nuclei broke through the internal limiting membrane (ILM) into the vitreous. For the orientation, two eyes (one eye of each animal) were selected from each group then enucleated and placed in 4% paraformaldehyde at 4 °C for 24 h, after that they were embedded in paraffin. Serial 5-μm sections (each separated by at least 30 μm) through the cornea and parallel to the optic nerve were prepared, stained with hematoxylin and eosin (H&E), and viewed by light microscopy (OLYMPUS Optical Co., Ltd., Japan), for the assessment of the retinal vasculature.

### Quantitative real-time PCR (qRT-PCR)

Using Trizol reagent (Invitrogen, Carlsbad, CA), total retinal RNA was isolated (four eyes from four mice from each group) according to the manufacturer’s instructions. Then RNA was reverse transcribed with reverse transcriptase (Promega, Madison, WI, USA) to generate cDNA, and the relative amounts of cyclin D1 transcript were determined by real-time quantitative PCR (qRT-PCR). The primers used were: 5′-TGC CAT CCA TGC GGA AAA TCG T-3′ and 5′-GCT CCT CGA CGA CGT TTA CCT T-3′ for Cyclin D1, and 5′-ATG GAT GAC GAT ATC GCT GCG C-3′ and 5′-TAC CTA CTG CTA TAG CGA CGC G-3′ for β-actin. The conditions of PCR were 94 °C for 30 min followed by 40 cycles at 95 °C for 20 s, 57 °C for 20 s, and 72 °C for 20 s. Measurements were performed three times independently.

### Western blot

Western blot was performed using the documented standard methods as we have done before [9]. Retinas (four eyes from four mice from each group) were dissected, immersed and homogenized in 0.1% triton X-100 extraction buffer (Solarbio, Beijing, China) containing phenylmethylsulfonyl fluoride (PMSF) and dithiothreitol (DTT), then the lysates were centrifuged to remove insoluble material, after which total retinal protein was extracted. Protein concentration was measured by a protein assay (Bradford Protein Assay; Bio-Rad, Hercules, CA, USA), and adjusted to allow equal total protein loading on the gels. Proteins were transfer to polyvinylidene fluoride (PVDF) membranes (Millipore, Billerica, MA, USA) using standard electroblotting procedures. Then membranes were blocked with 5% skim milk and then incubated with cyclin D1(1:500 in 5% BSA) (AC853, Beyotime Biotechnology, Jiangsu, China), or β-actin (1:5000 in 5% BSA) (Santa Cruz Biotechnology, Santa Cruz, CA, USA) primary antibodies at 4 °C overnight. Then membranes were incubated with horseradish peroxidase (HRP)-conjugated secondary antibodies (ZSGB-BIO, Beijing, China) at 37 °C for 1 h, and visualized by the ChemiDoc™ MP System (Bio-Rad, Hercules, CA, USA). Protein bands were quantified using Image-Pro plus 6.0 analysis software.

### Statistics

All data are presented as the means ± SD. Data were analyzed by the independent sample student’s *t*-test, or one-way ANOVA followed by LSD test. Differences were considered statistically significant when *p* < 0.05.

## Results

### Effect of RAPA on the inhibition of experimental RNV

**Fig. 1:**
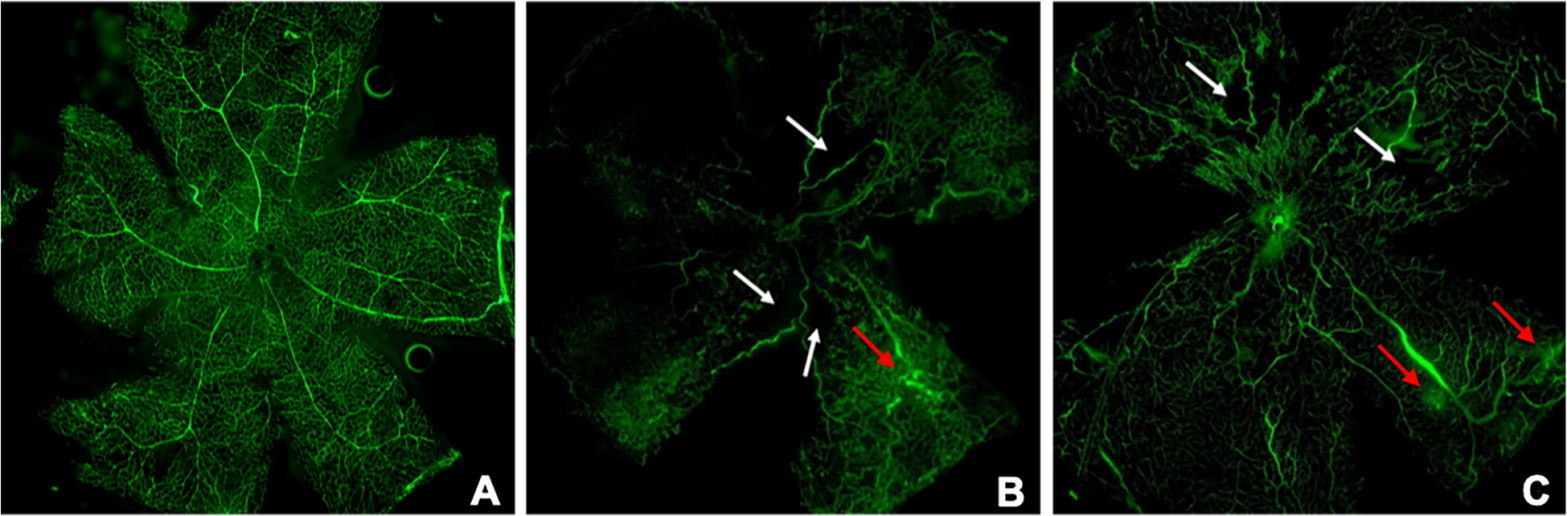
Vascular patterns in retinal flat mount after FITC–dextran perfusion (40×). **a** CON group (without treatment) retina shows well-perfused vessels and no neovascularization. **b** OIR group retina presents non-perfused area and neovascularization. **c** RAPA ameliorates the change in OIR retina. White arrow shows non-perfused areas. Red arrow shows neovascularization on retina

RNV was examined by FITC-dextran in retinal flat mounts at P17. Control group retinas had both superficial and deep vascular layers extended from optic nerve to periphery. Vessels in superficial retinal layers formed a fine radial branching pattern and in deeper retinal layers formed a polygonal reticular pattern (Fig. 1a). OIR group retinas were characterized by non-perfusion areas and neovascularization. Compared to OIR group, the ratio of non-perfused area to total retinal area was lower in RAPA group (0.018 ± 0.007 VS 0.059 ± 0.08; *t* = 8.780, *p* < 0.001), which demonstrates a strong inhibitory effect of RAPA on RNV in OIR model. (Fig. 1).

**Fig. 2:**
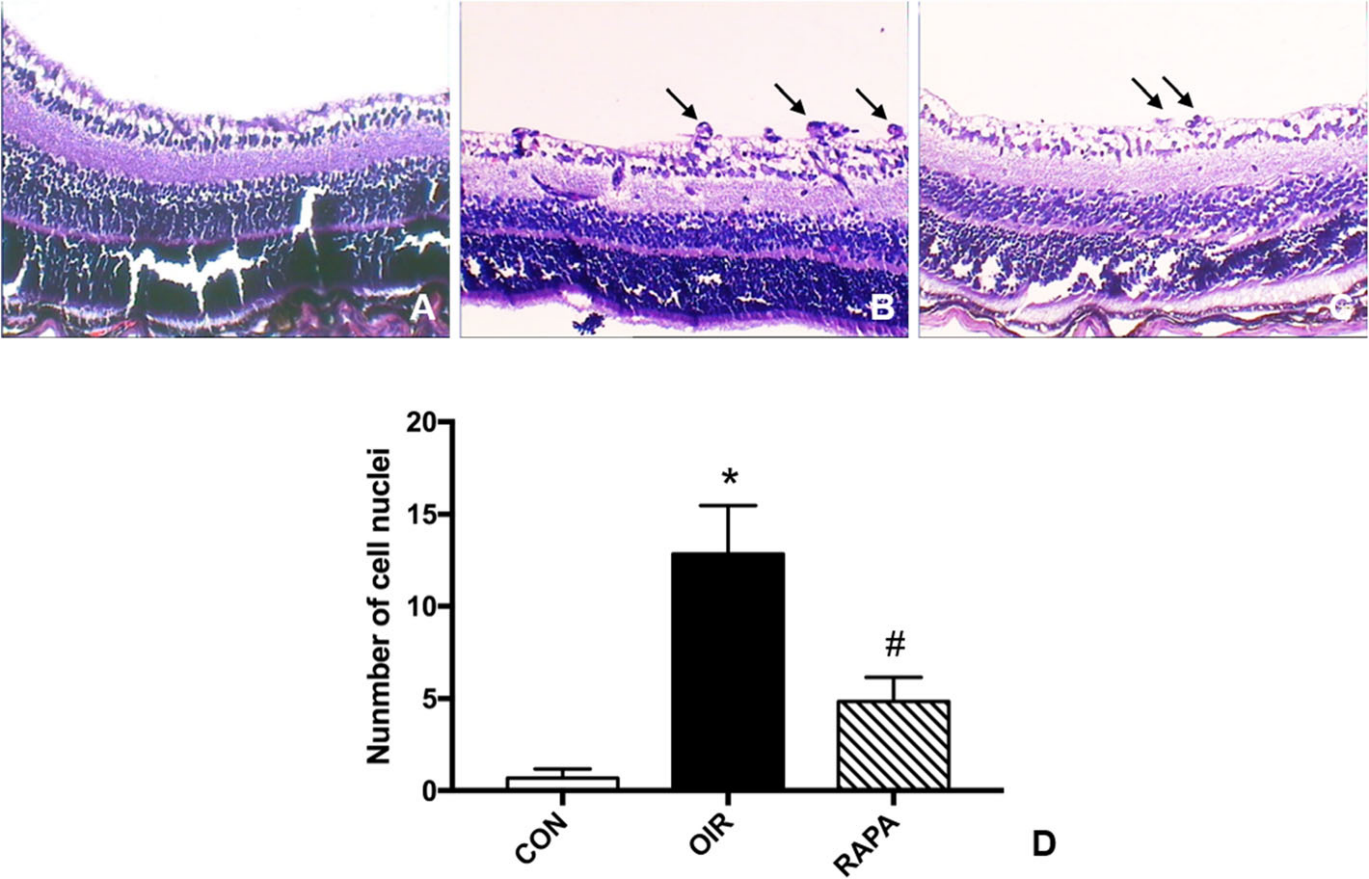
Light micrograph of retinal cross-sections from P17 mice. H&E 40×. **a** CON group (without treatment) retina. **b** OIR group retina. **c** RAPA group retina. **d** Graphs showing the average number of neovascular nuclei per retinal cross section. * *p* < 0.001, significantly different from the CON group; # *p* < 0.001, significantly different from the OIR group. Black arrow shows vascular cell nuclei broke through ILM

H&E stained retina sections are shown in Fig. 2, and the degree of RNV was quantified in serial 5-μm cross sections by counting the number of vascular cell nuclei on vitreous side of ILM. Large clusters of blood vessels were observed in OIR group, while fewer blood vessels were observed in RAPA group retinas. The average number of nuclei in CON, OIR and RAPA groups was 0.67 ± 0.52, 12.83 ± 2.64 and 4.83 ± 1.33, respectively (*F* = 76.463, *p* < 0.001) (Fig. 2).

### RAPA inhibits levels of cyclin D1

**Fig. 3:**
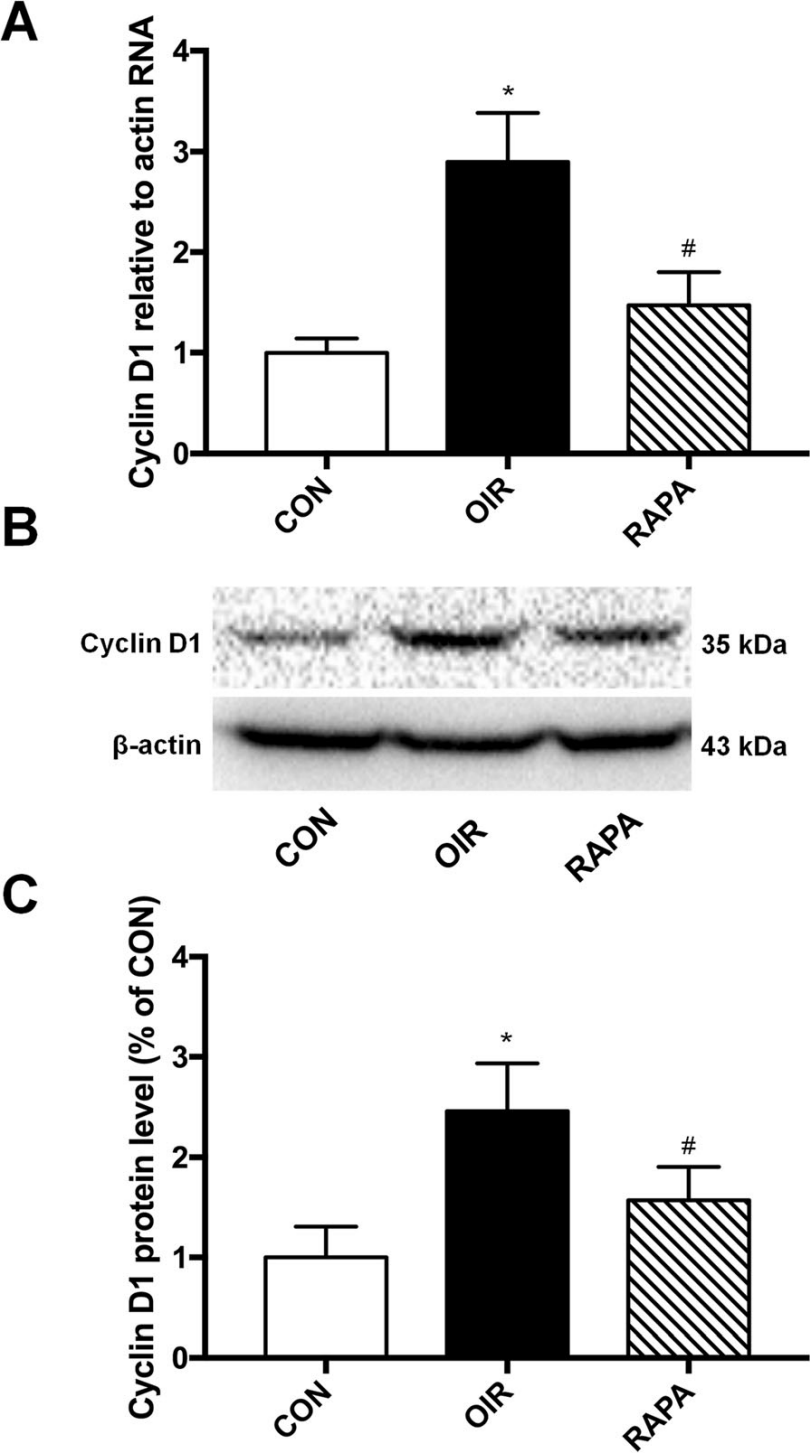
Effect of RAPA on mRNA and protein expressions of cyclin D1. **a** Expression of cyclin D1 mRNA. **b** Expression of cyclin D1 protein. **c** Graphs quantify expression of cyclin D1 protein. * *p* < 0.001, significantly different from the CON group; # *p* < 0.001, significantly different from the OIR group

Cyclin D1 is the most important positive regulatory factor of the cell cycle, and the effect of RAPA on the expression levels of both Cyclin D1 mRNA and protein were determined in the retina of OIR mice. The Cyclin D1 mRNA levels in the retina of OIR group was 289.8% ± 48.5% of CON, and RAPA treatment significantly reduced Cyclin D1 mRNA level (147.1% ± 33.1% of the control) (*F* = 22.641, *p* < 0.001). The protein expression level of Cyclin D1 demonstrated the same trend. Cyclin D1 protein level significantly increased in OIR group, and RAPA prevented the change, at least in part (246.3% ± 47.4 and 157.1% ± 33.4% of CON, respectively) (Fig. 3).

**Fig. 4:**
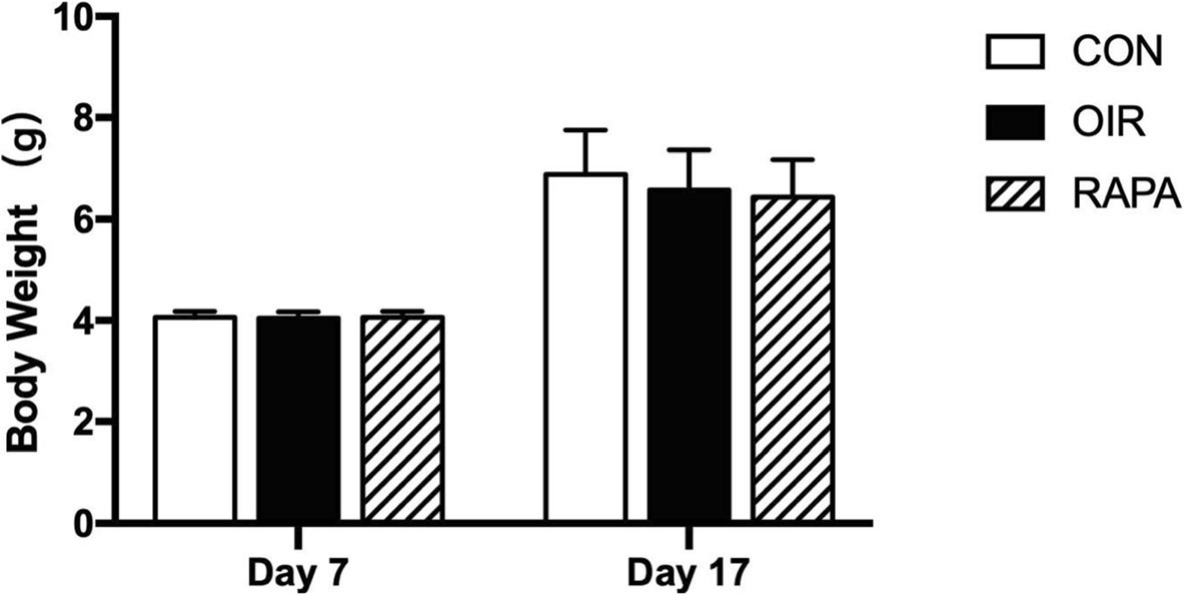
Comparison of body weight in CON group, OIR group and RAPA group, respectively. There was no significant difference body weight among CON group, OIR group and RAPA group on postnatal day 7(P7) (F=0.105, *p*=0.901). There was no significant difference body weight among CON group, OIR group and RAPA group on P17 (F=1.062, *p*=0.355)

Mice in the three groups all got weight (P17) and there was no significant difference in mean body weight among three groups, indicating that no obvious systemic side effect was associated with RAPA treatment (Fig. 4).

## Discussion

Retinal vascular ECs proliferation is a crucial step in the complicated process of pathological angiogenesis, thus, inhibition of vascular ECs proliferation demonstrates a potentially important treatment for blinding RNV diseases. The mouse model of OIR is the most widely used model for pathological angiogenesis resulting from ischemia, and it is a reliable method of quantifying retinal neovascularization [8, 10]. We, therefore, used the OIR model to evaluate the inhibitory effect(s) of RAPA on RNV, and explore the potential underlying mechanism.

The cell cycle is divided into four consecutive phases: gap 1 (G1), synthesis (S), and gap 2 (G2) phases; mitosis (M) phase (DNA segregation and cell division). Cyclin D1, as the most important positive regulatory factor of the cell cycle, could form active complexes with cyclin-dependent kinases (CDKs), either CDK4 or CDK6, which in turn phosphorylate the retinoblastoma protein (Rb) and regulate cell cycle transition from G1 to S phase. Overexpression of cyclin D1 results in activated CDK, rapid cell growth under conditions of restricted mitogenic signaling, bypass of key cellular checkpoints and ultimately cell proliferation [7, 11].

mTOR plays a key role in controlling cell growth and proliferation, regulating mitogens in mammalian cells and promoting cell cycle progression [12]. By activating downstream pathways, mTOR controls the translation of mRNA that encode proteins important for cell cycle progression, including cyclin D1 [12–14]. RAPA is a potent, naturally occurring mTOR inhibitor [15, 16], which prevents primary and metastatic tumor growth by antiangiogenesis [16].

Our data demonstrate that intraperitoneal injection of RAPA could reduce the number of retinal non-perfused areas and neovascular tufts. Moreover, our previous study [7] revealed that RAPA induced cell accumulation in the G1/G0 phase of retinal vascular ECs, which was in accordance with inhibition of ECs proliferation, and along with fewer cell nuclei on the vitreous side of ILM and downregulation of cyclin D1 in the retina. These results demonstrate that RAPA could inhibit retinal vascular endothelial cell proliferation and RNV, with the underlying mechanism of arresting the cell cycle in its transition from the G1- to S-phase via inhibition of cyclin D1 activity, which is different from current clinical used anti-VEGF drugs or glucocorticoids [17, 18].

## Conclusion

To sum up, RAPA can inhibit RNV by downregulating the expression of cyclin D1, which indicates its therapeutic potential in treating RNV-related diseases.

## Data Availability

All the data supporting our findings are provided in the manuscript and the appendix material.

## Abbreviations

AMD: Age-related macular degeneration
ARVO: Association for Research in Vision and Ophthalmology
BRVO: Branch retinal vein occlusion
CDKs: Cyclin-dependent kinases
CON: Control group
CRVO: Central retinal vein occlusion
DR: Diabetic retinopathy
DTT: Dithiothreitol
ECs: Endothelial cells
FITC: Fluorescein isothiocyanate
G1: Gap 1
G2: Gap 2
ILM: Internal limiting membrane
M: Mitosis
mTOR: mammalian target of rapamycin
OIR: Oxygen-induced retinopathy
P12: Postnatal day 12
PMSF: Phenylmethylsulfonyl fluoride
PVDF: Polyvinylidene fluoride
RAPA: Rapamycin
Rb: Retinoblastoma protein
RNV: Retinal neovascularization
ROP: Retinopathy of prematurity
